# The physiological role of fat body and muscle tissues in response to cold stress in the tropical cockroach *Gromphadorhina coquereliana*

**DOI:** 10.1371/journal.pone.0173100

**Published:** 2017-03-02

**Authors:** Szymon Chowański, Jan Lubawy, Ewelina Paluch-Lubawa, Marta Spochacz, Grzegorz Rosiński, Małgorzata Słocińska

**Affiliations:** 1 Department of Animal Physiology and Development, Faculty of Biology, Adam Mickiewicz University in Poznań, Poznań, Poland; 2 Department of Plant Physiology, Faculty of Biology, Adam Mickiewicz University in Poznań, Poznań, Poland; University of Cincinnati, UNITED STATES

## Abstract

Protective mechanisms against cold stress are well studied in terrestrial and polar insects; however, little is known about these mechanisms in tropical insects. In our study, we tested if a tropical cockroach *Gromphadorhina coquereliana*, possesses any protective mechanisms against cold stress. Based on the results of earlier studies, we examined how short-term (3 h) cold (4°C) influences biochemical parameters, mitochondrial respiration activity, and the level of HSPs and aquaporins expression in the fat body and leg muscles of *G*. *coquereliana*. Following cold exposure, we found that the level of carbohydrates, lipids and proteins did not change significantly. Nevertheless, we observed significant changes in mitochondrial respiration activity. The oxygen consumption of resting (state 4) and phosphorylating (state 3) mitochondria was altered following cold exposure. The increase in respiratory rate in state 4 respiration was observed in both tissues. In state 3, oxygen consumption by mitochondria in fat body was significantly lower compared to control insects, whereas there were no changes observed for mitochondria in muscle tissue. Moreover, there were cold-induced changes in UCP protein activity, but the changes in activity differed in fat body and in muscles. Additionally, we detected changes in the level of HSP70 and aquaporins expression. Insects treated with cold had significantly higher levels of HSP70 in fat body and muscles. On the other hand, there were lower levels of aquaporins in both tissues following exposure to cold. These results suggest that fat body play an important role in protecting tropical insects from cold stress.

## Introduction

Temperature is an important abiotic variable that can determine the ecological niche of a species. Temperature has significantly influenced the distribution of living organisms in different geographical environments. Insects inhabit almost all geographical zones from the tropics to the polar regions. They are absent only in the depths of the oceans [1]. The presence of insects in areas with varied temperature conditions is possible due to the development of adaptations that permit them to avoid the harmful effects of hot and cold temperatures. Although there are many strategies that insects have evolved to cope with low temperatures, these strategies can be divided into two main groups: freeze avoidance and freeze tolerance. Both strategies may involve the development of morphological, cellular and behavioural adaptations [2–7].

Adaptations to cold stress in insects can be distinguished based on the activation of main metabolic pathways [3,7–9], the accumulation of carbohydrates, polyols and free amino acids in haemolymph [10–13], and the synthesis of different proteins, such as aquaporins (AQPs) [14–17], ice nucleating agents (INAs) [3,6,18–20] antifreeze proteins (AFPs) [13,21–23] and heat shock proteins (HSPs) [24–26]. Increased activity of glycolysis, gluconeogenesis and pentose phosphate pathway enzymes is involved in the synthesis of cryoprotectant agents, such as glycerol, sorbitol, glucose, trehalose and proline, which are released into haemolymph [8]. In the fly, *Sarcophaga bullata*, increases in phosphoenolpyruvate carboxykinase [27], glucose 6-phosphatase and fructose 6-phosphatase activity was observed during glycolysis and gluconeogenesis in response to rapid cold hardening [9]. Moreover, in insects that store glycogen in tissues, exposure to cold stress increased the activity of glycogen phosphorylase [5]. High concentrations of cryoprotectants in haemolymph increase osmotic potential so that freezing can be avoided. The presence of cryoprotectants in haemolymph lowers the supercooling point, which prevents haemolymph from freezing [28,29]. In the gall moth *Epiblema scudderian*, body fluid can be supercooled to -40°C. Essential is also the transfer of water from cells into the intercellular space. For this process aquaporins are crucial. These membrane proteins, which form channels for water transport, increase membrane permeability for water and glycerol (aquaglyceroporins). Thus, they play a role in the regulation of the osmotic potential of extra- and intracellular fluid, which influences the efficiency of ice crystal growth. Insects experiencing low temperatures have also exhibited changes in the level of AQP expression [30]. The role of AQPs in cold resistance was confirmed by Izumi et al. [31] and Zelenina et al. [32], who showed that when AQPs are blocked by mercury or copper, the resistance of insect cells to cold stress decreases. Changes in AQPs expression in response to cold stress have been observed in the larvae of the fruit fly *Eurosta solidaginis* [15] and in the adult cockroach *G*. *coquereliana* [30].

Low temperatures also influence the activity of other enzymes and proteins, such as protein kinases that regulate i.a. metabolic activity of mitochondria [33], antioxidant enzymes (catalase, dismutase) [34,35], and HSPs [25,36,37]. Increases in different HSP proteins vary between species. For example, in pupa of the fly *Delia antiqua*, increases in HSP 60 and HSP 70 have been observed. On the other hand, in the fly *S*. *bullata*, the primary HSPs proteins that increased in response to low temperatures were HSP 23, HSP 70 and HSP 104. In the cockroach *G*. *coquereliana*, low temperatures can decrease the level of certain subtypes of HSP proteins and also lead to the appearance of new subtypes of HSP proteins [30]. Moreover, cold stress can lead to changes in immune system activity. Some immune system variables that can be affected by cold in the burring beetle *Nicrophorus vespilloides* include total haemocyte count, phenoloxidase activity, and the phagocytic ability of haemocytes [38,39].

Cold temperatures also affect mitochondrial activity. In the diapausing larvae of *E*. *solidaginis* that experience temperatures as low as -15°C, the activity of mitochondrial enzymes, such as citrate synthase, glutamate dehydrogenase, and NAD-isocitrate dehydrogenase, was lowered by 50% compared to insects experiencing temperatures greater than 15°C [40]. Similarly, mitochondrial enzymes involved in fatty acid synthesis (malic enzyme) and fatty acid oxidation (carnitine palmitoyltransferase, 3-hydroxyacyl-CoA dehydrogenase, thiolase) have been shown to be suppressed during the winter [41]. Nevertheless, reduced mitochondrial metabolism may be the result of a decrease in the number of mitochondria, as has been shown in *E*. *solidaginis* [42]. These authors also showed that the wintering (-15°C) larvae of *E*. *solidaginis* had 60% lower oxygen consumption than larvae collected during the summer (15°C). Moreover, different stressors may lead to different changes in the primary substrates used by mitochondria during ATP synthesis.

The aim of this study was to determine the effect of low temperatures on some aspects of physiology of a tropical insect species, the Madagascar hissing cockroach *Gromphadorhina coquereliana*, and whether such mechanisms as changes in mitochondrial activity and changes in proteins, lipids and carbohydrates levels in tissues or the presence of specific proteins connected to responses to cold temperatures, such as aquaporins and HSPs may be involved in response to cold stress. Currently, information about adaptation to cold stress is abundant in insects from temperate and polar climates, which experience substantially cold temperatures annually. We chose to study the Madagascar hissing cockroach because of its natural environment. This insect lives in the diverse subtropical climate of Madagascar, where it tends to be colder at higher elevations and particularly dry and warm in the southern and western regions. The highest temperatures occur during the month of December, with average daily highs of 28°C and lows of 17°C. July is the coolest month, with average daily temperatures ranging from 9°C to 21°C [43,44]. The average minimum temperature in this month is approximately 10°C, and the lowest registered temperature was 1°C. Nevertheless, during the last 10 years, the temperature has dropped below 5°C approximately 100 times (in the capital Antananarivo), and this temperature occurred only at night for a maximum of 3–4 h [44].

We addressed whether tropical insects, which are occasionally affected by low temperatures for short periods of time, react to cold stress similar to insects from temperate and polar regions. Our previous studies have shown that, in response to long-term cold stress, protein levels increase, and glycogen content decreases in fat body. Moreover, we have also shown that cold temperatures lead to changes in the expression of HSPs and AQPs. Following cold treatment, the quantity of AQPs in fat body increased. AQPs increased whether insects were exposed to only 8 h of cold temperatures or were repeatedly exposed to cold temperatures. We found that cold temperatures lead to the appearance of new isoforms of HSPs [30]. In previous research, we have exposed the insects to long periods of cold stress; however, in the natural environment of Madagascar, low temperatures affect cockroaches for a maximum of 2–4 h. What is important, our preliminary data indicate, that low temperature (4°C) did not increase the mortality of *G*. *coquereliana* after 3, 8 and 24 hours of cold treating with single and triple repetition of cold stress and did not induce chill coma. In the present study, we treated insects with cold temperatures for 3 h and collected samples of fat body and leg muscle tissue immediately following cold exposure. Our goal was to determine how physiological processes change in the immediate response of *G*. *coquereliana* to short-term cold temperatures. We decided to check the biochemical parameters like glycogen, lipids and protein content in tissues which may indicate changes in metabolic processes. Additionally we measured levels of aquaporins and HSP, proteins involved in response to cold stress [45]. Moreover, we supposed, that activity of uncoupling protein 4 (UCP4) should increase during cold stress. There may be two reasons for that, one is that UCP4 are involved in reduction of free radicals [46,47] or in generation of heat [48]. We supposed that the processes involved in generation of heat are one of defence mechanism during short-term cold. Uncoupling protein (UCP1-thermogenin) takes part in energy dissipation in mammals mitochondria and it was also found in insects, so its increased activity under cold stress suggests thermogenic role of UCP in insect. Moreover, we supposed that increased UCP activity is related with diminishing of oxidative stress evoked by cold stress. Disruption of ATP synthesis and role of mitochondria in energy homeostasis during stress low temperature was shown previously by Colinet [49].

## Materials and methods

### 2.1 Insects

Cockroaches (*Gromphadorhina coquereliana*) were obtained from continuous colony reared under laboratory conditions at 28°C and approximately 65% relative humidity under a 12 h light/12 h dark cycle in Department of Animal Physiology and Development, AMU in Poznań. Food (lettuce, carrots, and powdered milk) and water were provided *ad libitum*. Only adult male individuals of approximately 5.8 cm (± 0.31 cm) in size and a weight of 5.3 g (± 0.48 g) were used for experiments. During the experiment, insects were placed in a plastic boxes in an air-conditioned cold room with a stable temperature of 4°C and approximately 65% (± 2%) humidity recorded by a Thermo-Hygro-Station (TFA-Dostmann, Germany). The animals (3 to 4 individuals) were kept in plastic boxes (15 x 30 x 20 cm) with carrots for food.

The insects were treated with a low temperature (4°C) for 3 h. Samples were collected immediately after cold treatment. In all experiments, *G*. *coquereliana* were anaesthetized by submerging them under water for 20 min. After anaesthesia, insects were injected with 300 μl of anticoagulant buffer (AC) containing 69 mM KCl, 27 mM NaCl, 2 mM NaHCO_3_, 30 mM sodium citrate, 26 mM citric acid and 10 mM EDTA, pH 7.0, and prepared according to the modified procedure of [50]. The injection of anticoagulant was performed for purification of fat body and muscles from haemolymph. *G*. *coquereliana* were injected under their last pair of legs using a Hamilton syringe and were left for 5 min to allow the AC to spread throughout the insect body. After that the insects were decapitated and legs were cut off. The legs were wash with saline (139 mM NaCl, 5 mM KCl, 4 mM CaCl_2_) to remove rest of haemolymph. For muscles isolation only femur was used. Next the integument was cut with microsurgical scissors around whole insect body and the abdominal part of cuticle was removed and next the fat body was intensively washed with saline. Malpighian tubules and tracheas were removed from isolated tissues with microsurgical tweezers.

### 2.2 Analysis of glycogen content in tested tissues

The isolation of glycogen was conducted according to the procedure of Van Handel (1965). After tissue (fat body and muscles of legs) isolation, samples were placed in Eppendorf tubes and were dried to a stable weight at 60°C under a vacuum (-0.9 atm). The dry mass of samples was measured, and the samples were then lysed in 30% KOH for 15 min at 90°C. Following tissue lysis, a saturated solution of Na_2_SO_4_ and 70% ethanol (8:1:16; v/v/v) was added to precipitate the glycogen. Next, the sample was centrifuged at 10,000 x g for 5 min. The supernatant was removed, and the pellet was washed twice with 70% ethanol. The acquired pellet was then dissolved in water and shaken for 10 min at 80°C. The glycogen content of the solution was then measured using the phenol-sulphuric acid method of Dubois et al. [51]. Oyster glycogen (Sigma-Aldrich) was used as a standard.

### 2.3 Evaluation of the total lipids in tested tissues

For control or tested insects, 5–10 mg of fat body or 25–30 mg of muscle tissue was collected in Eppendorf tubes. After drying to a stable mass at 60°C under a vacuum (-0.9 atm), the dry weight of the sample was measured. The isolation of lipids from tissues was performed according to Folch’s method [52]. The tissue was homogenized in a mixture of chloroform and methanol (2:1, v/v) for 2 min and centrifuged at 10,000 x g for 10 min. The supernatant was transferred to a new tube and washed three times with 0.29% NaCl. Finally, the solvent was evaporated at 30°C under a vacuum (30°C, -0.9 atm). The pellet was dissolved again in a chloroform and methanol mixture (2:1, v/v). Aliquots of the mixture were taken, and lipid content was measured gravimetrically after the evaporation of the solvent.

### 2.4 Determination of protein content in fat body and muscle tissue

The collected tissue was dried to a stable mass at 60°C under a vacuum (-0.9 atm), and the dry weight of the sample was measured. Next, the samples were homogenized in a saline solution (139 mM NaCl, 5 mM KCl, 4 mM CaCl_2_) and centrifuged at 5,000 x g for 10 min. The supernatant was used to determine the content of proteins in the soluble fraction. The measurement was conducted with a Direct Detect^®^ Infrared Spectrometer (Merck Millipore). A total of 2 μL of supernatant was placed on the PTFE membrane and was left to dry for 3 min. The protein content was then measured. Bovine serum albumin (Merck Millipore) was used as a standard.

### 2.5 Immunodetection

A fraction of the soluble proteins was resuspended in sample buffer (139 mM NaCl, 5 mM KCl, 4 mM CaCl_2_). The concentration of proteins in samples was determined with a Direct Detect^®^ Infrared Spectrometer (Merck Millipore). SDS-PAGE electrophoresis (using a 5% polyacrylamide stacking gel and 14% polyacrylamide resolving gel) and Western blotting were performed as previously described [53]. For each line was loaded 50 μg of proteins suspended in Laemmli sample buffer (BioRad) boiled for 5 min. Electrophoresis was performed for 1h with voltage 200 mV in electrophoretic buffer (2.5 mM Tris-HCl, 0.02 M glycine, 0.01% SDS) with electrophoresis apparatus (BioRad). Electrotransfer was carried out for 70 minutes in electrotransfer buffer (20% methanol, 0.02% SDS, 20 mM Tris-HCl, 0.15 M glycin) at current 60 mA with apparatus to electrotransfer (Biorad). Mouse polyclonal primary antibodies against HSP70 (Agrisera, AS09 592) at a 1:2,000 dilution (incubation for 75 min at room temp.) with goat anti-mouse horseradish peroxidase-conjugated secondary antibodies (Agrisera, AS09 602) at dilutions of 1:25,000 (incubation for 75 min at room temp.) were used. For aquaporins, rabbit polyclonal primary antibodies against *Arabidopsis thaliana* aquaporins (PIP2: PIP2;1, PIP2;2, PIP2;3; Agrisera, AS09 491) at a dilution of 1:1,000 (incubation for 1 h at room temp.) and goat anti-rabbit horseradish peroxidase-conjugated secondary antibodies (BioRad, 166-2408-MSDS) at a dilution of 1:20,000 (incubation for 1 h at room temp.) were used. Rabbit antibodies against α-tubulin (Sigma-Aldrich, T-9026) at a 1:500 dilution were used as a loading control. The protein bands were visualized using Amersham ECL. The density of the protein bands on the blots was measured using Biostep GelixOne G230 software.

### 2.6 Isolation of mitochondria

Muscle mitochondria were isolated using previously described methods [54]. Isolation was performed in a medium containing 100 mM KCl, 50 mM Tris-HCl (pH 7.4), 1 mM K_2_HPO_4_, and 0.2% bovine serum albumin (BSA). Tissues were homogenized in a glass-teflon homogenizer. For one sample, tissues from 3 individuals were pooled. The homogenate was then centrifuged for 10 min at 500 x g. The acquired supernatant was centrifuged for 10 min at 10,000 x g. The final pellet containing mitochondria was resuspended in medium containing 0.2 M mannitol, 0.1 M sucrose, 10 mM Tris-HCl (pH 7.4), and 0.1 mM EDTA. The pellet was then stored on ice for subsequent assays.

The mitochondria from fat body were isolated according to the modified procedure of Sujak [55]. Isolation was conducted in a medium containing 0.25 M sucrose, 1 mM EDTA, 5 mM Tris-HCl and 1% BSA. The contents were then homogenized in a glass-teflon homogenizer. The homogenate was then centrifuged for 10 min at 800 x g, and the collected supernatant was centrifuged at 12,000 x g for 10 min. The acquired pellet was then washed twice in a medium containing 0.25 M sucrose, 1 mM EDTA, and 5 mM Tris-HCl and was centrifuged at 8,000 x g for 10 min. The final pellet containing mitochondria was resuspended in the same medium. All of the aforementioned procedures were performed at 4°C. Mitochondrial protein concentrations were determined using a Direct Detect^®^ Infrared Spectrometer (Merck Millipore).

### 2.7 Measurement of oxygen consumption by mitochondria

Oxygen consumption of *G*. *coquereliana* muscle and fat body mitochondria was measured at 25°C with a Clark-type electrode (Oxytherm, Hansatech) in 0.8 ml of the incubation medium containing 0.2 M mannitol, 75 mM sucrose, 10 mM KCl, 0.1 mM EDTA, 10 mM K_2_HPO_4_, and 10 mM Tris-HCl (pH 7.4). Succinate (10 mM) or pyruvate (10 mM) plus malate (10 mM) were used as oxidizable substrates for the measurements of fat body and muscle mitochondrial respiration. To induce uncoupling protein 4 (UCP4) activity-mediated respiration, measurements in the presence of palmitic acid (15 μM) were performed. Palmitic acid-induced UCP4 activity was inhibited by the addition of 2 mM GTP. To exclude the activities of the ATP/ADP antiporter and ATP synthase, carboxyatractyloside (1.5 μM) and oligomycin (1 mg per 1 mg of mitochondrial protein) were used for resting (state 4) measurements. The respiratory control ratio (RCR) was calculated as the ratio of state 3 to state 4. Values for O_2_ uptake are presented in nM O_2_*min^−1^*mg protein^−1^.

### 2.8 Statistical analysis

All data are presented as the mean values ± SD of the number of replicates (*n*) indicated. The statistical significance of differences between values of control insects and those exposed to cold stress were determined using a Student's *t*-test. The statistical analyses were performed using GraphPad Prism software. Differences were considered statistically significant if *p*≤0.05 (*), *p*≤0.01 (**), or *p*≤0.001 (***).

## Results

### 3.1 Effects of low temperature on free sugars, glycogen, total lipids and protein content

**Fig 1:**
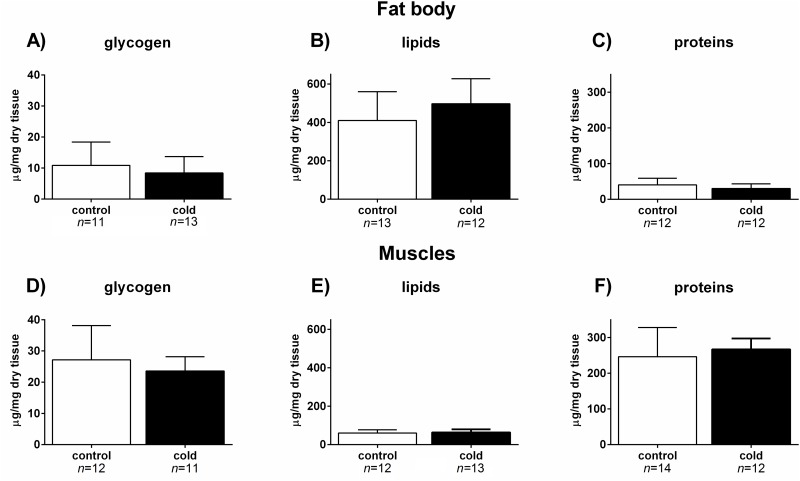
Changes in glycogen, lipid and protein content in the fat body and leg muscles of *G*. *coquereliana* cockroaches. Control—insect not exposed to cold stress. Cold—insect stressed for 3 h by cold (4°C). Data represent mean value ±SD. Significant differences from the respective control value are indicated by asterisks: *p*≤0.05 (*), *p*≤0.01 (**), or *p*≤0.001 (***). Student's *t*-test.

Exposure to cold stress (4°C for 3 h) did not lead to significant changes in the biochemical parameters of tested tissues. Nevertheless, there was a slight tendency for the glycogen content in both fat body and muscle tissues to decrease following cold exposure. The amount of glycogen was lowered by 24% in the fat body and by 12% in the muscles of insects following low temperature treatment compared to those of the control insects, but the changes were not statistically significant (*p =* 0.3600; *t =* 0.9349 and *p =* 0.3323; *t =* 0.9924), respectively for fat body and muscles (see Fig 1A and 1D). Moreover, we observed also increase in content of total lipids in fat body by 20% (*p =* 0.1381; *t =* 1.536) and muscle tissues by 5% (*p =* 0.6320; *t =* 0.4854) (Fig 1B and 1E). Determination of fraction of soluble proteins also did not show significant changes in these parameters (Fig 1C and 1F). In fat body the level of soluble proteins was 25% lower in insects treated with cold than in control insects (*p =* 0.1295; *t =* 1.575), whereas in muscles the cold caused increase in protein content by 8% (*p =* 0.4153; *t =* 0.8290).

### 3.2 The influence of low temperatures on the expression of HSP and AQP

**Fig 2:**
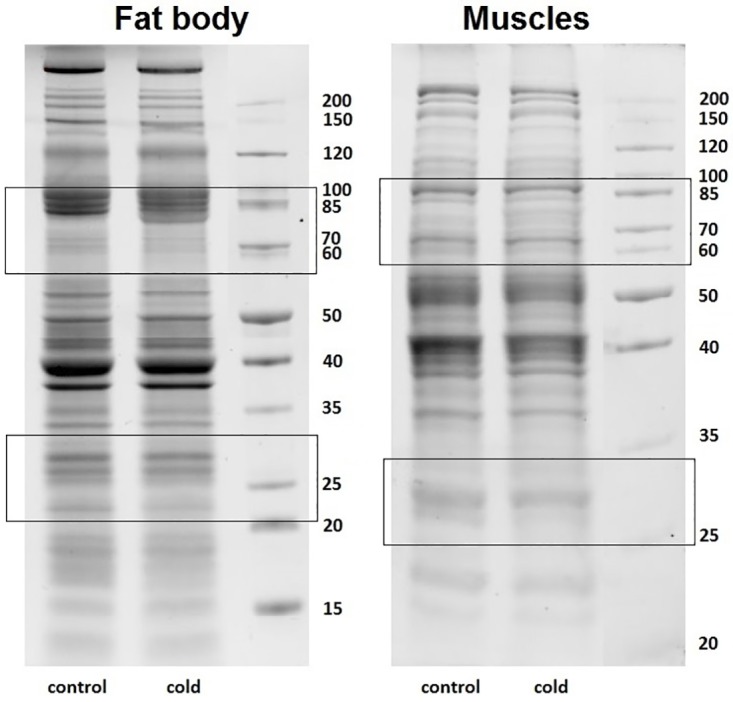
Protein profiles of the fat body (A) and leg muscles (B) of *G*. *coquereliana cockroaches*. The frames indicate bands of proteins that show significant changes in protein levels.

**Fig 3:**
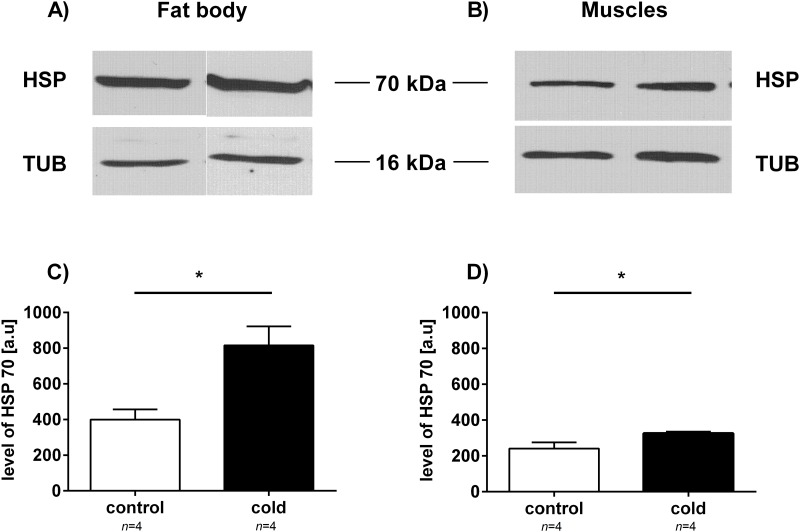
Changes in HSP 70 levels in the fat body and leg muscles of *G*. *coquereliana cockroaches*. Control—insect not exposed to cold stress. Cold—insect stressed for 3 h by cold (4°C). A) Immunological detection of HSP 70. Each lane had 50 μg of the soluble protein fraction loaded. The results are representative of at least 4 independent experiments. B) The average (±SD) HSP 70 protein expression level in tested tissues. The protein bands were digitally quantified using Biostep GelixOne G230 software. Significant differences from the respective control value are indicated by asterisks: *p*≤0.05 (*), *p*≤0.01 (**), or *p*≤0.001 (***). Student's *t*-test.

**Fig 4:**
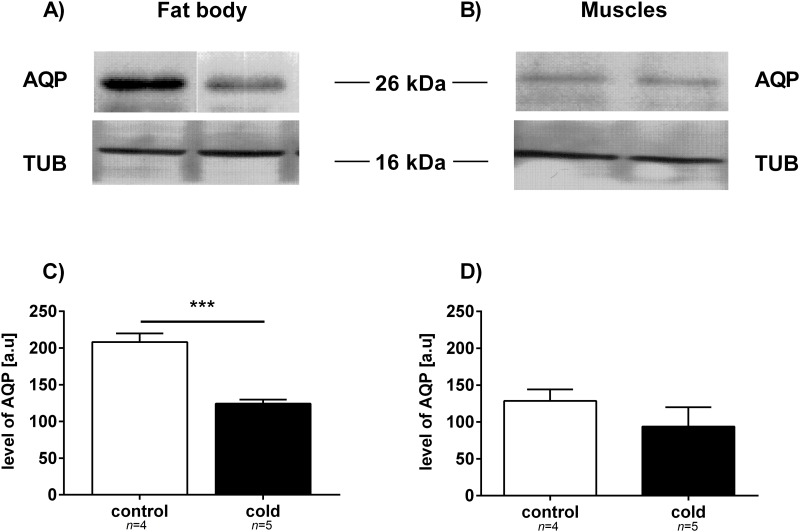
Changes in the level of AQP-like proteins in the fat body (A and C) and leg muscles (B and D) of *G*. *coquereliana* cockroaches. Control—insect not exposed to cold stress. Cold—stressed for 3 h by cold (4°C). A and B) Immunological detection of AQP-like proteins. Each lane had 50 μg of the soluble protein fraction loaded. The results are representative of at least 4 independent experiments. C and D) The average (±SD) AQP-like protein expression levels in tested tissues. The protein bands were quantified digitally using Biostep GelixOne G230 software. Significant differences from the respective control value are indicated by asterisks: *p*≤0.05 (*), *p*≤0.01 (**), or *p*≤0.001 (***). Student's *t*-test.

Total protein levels did not significantly change following cold exposure. We detected some differences in the protein profiles of control and cold-exposed insects (Fig 2A and 2B). Increases in protein levels of approximately 70 kDa were observed in the fat body and muscle tissues of the cockroach *G*. *coquereliana*. Additionally, the level of some proteins with lower molecular weights, approximately 60 kDa and 30 kDa in fat body tissue and approximately 30 kDa in leg muscle tissue, decreased in response to cold temperatures. Moreover, we investigated the effect of cold stress on the HSP and AQP levels of the fat body and muscle tissues of *G*. *coquereliana*. Immunological detection indicated that there were higher levels of HSP after 3 h of cold exposure (Fig 3A–3D). Specifically, there were increases of approximately 100% and 30% in the levels of HSP in fat body and muscle tissues, respectively. In both cases, changes were statistically significant (*p =* 0.0005; *t* = 6.895 and *p =* 0.0033; *t* = 4.708, respectively for fat body and muscles). On the other hand, the level of AQP in fat body tissue markedly decreased by approximately 40% (*p* = 0.0001; *t* = t = 14.380) following cold exposure (Fig 4A and 4C). The level of AQP in muscle remained unchanged in response to cold temperatures (*p =* 0.0533; *t* = 2.321) (Fig 4B and 4D).

### 3.3 Mitochondria respiratory activity and coupling parameters under cold stress

**Fig 5:**
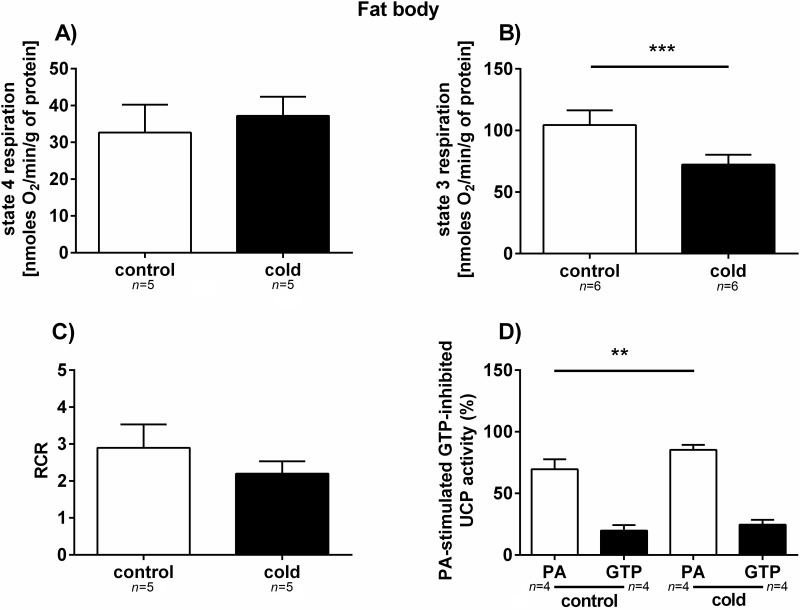
Bioenergetic parameters of mitochondria isolated from the fat body tissue of *G*. *coquereliana*. (A-B) Respiration was measured in the presence of 10 mM succinate and in the absence (state 4 respiration) or presence of 400 μM ADP (state 3 respiration). C) RCR refers to respiratory control ratio. D) Changes in the UCP activity of the fat body of *G*. *coquereliana* cockroaches following cold exposure. UCP activity was measured in isolated mitochondria in the presence of succinate palmitic acid (PA, an activator of UCP) and GTP (an inhibitor of UCP). Data represent mean value ±SD. Statistical significance is indicated by either *p*≤0.05 (*) or *p*≤0.01 (**), or *p*≤0.001 (***). Student's *t*-test.

**Fig 6:**
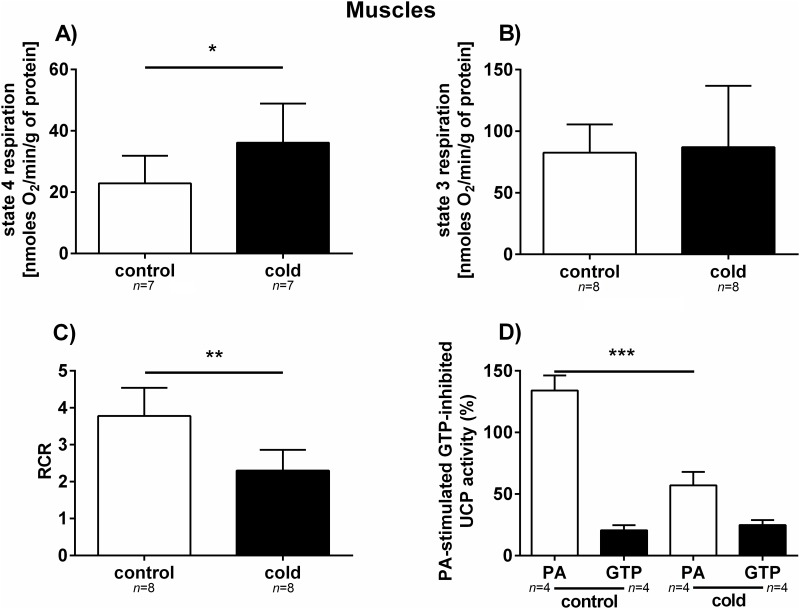
Bioenergetic parameters of mitochondria isolated from the muscle tissue of *G*. *coquereliana*. (A-B) Respiration was measured in the presence of pyruvate (10 mM) plus malate (10 mM) and in the absence (state 4 respiration) or presence of 400 μM ADP (state 3 respiration). C) RCR refers to respiratory control ratio. D) Changes in the UCP activity in the fat body and muscles of *G*. *coquereliana* following cold exposure. UCP activity was measured in isolated mitochondria in the presence of pyruvate, palmitic acid (PA, an activator of UCP) and GTP (an inhibitor of UCP). Data represent mean value ±SD. Statistical significance is indicated by either *p*≤0.05 (*) or *p*≤0.01 (**), or *p*≤0.001 (***). Student's *t*-test.

The activity of the cytochrome pathway was measured in mitochondria isolated from the fat body and muscle tissues of the control and cold-treated cockroaches. The oxygen consumption of resting (state 4) and phosphorylating (state 3) mitochondria, as well as coupling parameters, such as the respiratory control ratio (RCR), were measured. The increase of the respiratory rate of state 4 respiration was observed in both analysed tissues. However, only the increase in the respiratory rate of muscle (approximately 60%) was statistically significant (*p* = 0.0443; *t* = 2.246) (Figs 5A and 6A). In state 3, oxygen consumption by fat body mitochondria from cold-stressed cockroaches was reduced by approximately 35% compared to control insects (*p =* 0.0003; *t* = 5.515), whereas in muscle mitochondria, no statistically significant changes in phosphorylation were observed (*p =* 0.8224; *t* = 0.2288) (Figs 5B and 6B). The RCR ratio dropped in both muscle and fat body tissues; however, this difference was only statistically significant in muscle mitochondria (*p* = 0.0006; *t* = 4.415) (Fig 6C). The UCP activity in fat body and muscle tissue was assessed through the stimulation of this protein with palmitic acid (PA) and its inhibition with GTP. The activity of UCP in muscle mitochondria was much higher than in fat body mitochondria; however, 3 h of cold exposure significantly lowered UCP activity (*p*<0.0001; *t* = 9.372) (Fig 6D). In addition, the activation of UCP by PA in fat body increased significantly in response to cold stress (*p* = 0.0138; *t* = 3.439), what is an opposite effect to this observed in muscles (compare Figs 5D and 6D).

## Discussion

*G*. *coquereliana*, although it is a tropical insect, seems to be quite well insensitive to low temperature (4°C). Our preliminary studies indicate that cold (4°C) did not increase the mortality of *Gromphadorhina coquereliana* after 3, 8 and 24 hours of cold treating with single and triple repetition of cold stress. It is interesting the more that in natural environment this species may be exposed to low temperature for short time (2–4 hour per day). Moreover, on Madagascar temperature decreases to 3–4°C only few times per year. Thus, our recent study focuses primarily on the biochemical bases of the response of insects to low temperatures. Specifically, we examined the changes in physiological processes which may be involved in response of *G*. *coquereliana* to cold temperatures. Changes in protein, lipid and carbohydrate levels and energy management by mitochondria that are associated with cold stress were analysed in fat body and leg muscles of *G*. *coquereliana*.

The analysis indicated that there were not significant changes in overall lipid, carbohydrate and protein levels in fat body and in muscle tissue after cold stress (Fig 1A–1F), what is opposite to result obtained i.a. by Overgaard et al. [56] which showed that significant changes in metabolites rate are observed even after 2-hours of cold stress Similarly, Tamang et al. [57] demonstrated that short-term cold stress induce change in metabolite level like trehalose and proline concentration in haemolymph of *Drosophila immigrans*. In case of this cockroach species it is possible that 3 h of cold stress may be too short period to induce changes in biochemical parameters. Hence cockroaches may rely on other mechanisms/metabolic changes protecting them from cold stress. Our previous studies [30], where we tested influence of low temperature for 8 h, also did not indicate significant changes in glycogen and lipids level in fat body, but in case of glycogen tendency of changes was similar. Total protein content did not change as well; nevertheless there were observed changes in protein level at approximately 70–85 kDa and 25–30 kDa (Fig 2A and 2B). At the molecular level, the number of genes and proteins are known to play a role in insect stress. Cold exposure may induce changes in cell membrane proteins and enzymes related to insect energy metabolism (e.g., fatty acid metabolism, mitochondrial function, polyol synthesis or other cryoprotectants) [3,40,58]. Thus, the augmented synthesis of proteins involved in insect protection as well as the reduction of proteins associated with lowered metabolism might take place in *G*. *coquereliana*. For better understanding of response of tested cockroach species to cold further studies are needed, especially analysis of level of such compounds as polyols, carbohydrates and free amino acids in haemolymph, which play an important role in fast response to cold [59].

One of the most studied proteins involved in thermal responses are heat shock proteins (HSP), which consist of molecular chaperons that refold denatured proteins during periods of stress [60]. Such expression levels of HSPs due to cold stress have been observed in *Sarcophaga crassipalpis* [61], *Drosophila melanogaster* [62,63] and the Colorado potato beetle, *Leptinotarsa decemlineata* [64], in response to long-term cold exposure and during recovery time. Nevertheless, increase of HSPs levels was also observed in short-term cold exposure. Airaksinen et al. [45] indicated that level of HSP70 in zebrafish ZF4 cells increased significant after 4 h from start-point of cold stress. Foster et al. [65] showed, that level of mRNA of HSP70 and HSP 40 increased over 15-times in 30 min after 1 h exposure to cold stress in the central nervous system of *Lymnaea stagnalis*. The function of HSPs may be different during recovery time (neutralization of damages after cold stress) and during stress (protection against damages during cold stress). Our immunological studies showed that there were large increases (*p*≤0.05) in HSPs after 3 h of cold exposure in both of the analysed tissues of *G*. *coquereliana* (Fig 3A–3D). These results were similar to those observed in cockroaches after 8 h of cold stress and 8 h of recovery time [30]. The significant elevation of HSP level indicates that tropical insects are capable of rapidly responding to temperature fluctuations and suggests that HSPs play a protective role during stressful conditions.

Different changes were observed for aquaporins, water channels that play critical roles in protecting cells from osmotic damage by redistributing water and small solutes across cell membranes. The role of aquaporins in desiccation, osmoregulation and cold hardiness in insects has been previously reviewed by Cohen [66]. In this study, we have shown that AQP level in *G*. *coquereliana* does not change in muscle tissue. On the other hand, the AQP levels in the fat body tissue of *G*. *coquereliana* dropped drastically (*p*≤0.001) after 3 h of cold exposure (Fig 4). Previously, we found that the AQP in *G*. *coquereliana* is an aquaglyceroporin like-protein named AQP3 [30]. AQP3 is responsible for glycerol and water movement and was immunologically identified in most larval tissues of the Antarctic midge *Belgica antarctica*. Upregulation of AQP3 in *B*. *antarctica* was observed in response to dehydration [67]. In contrary, the AQP in the freeze-tolerant gall fly *E*. *solidaginis* is most closely related to AQP1. This protein is barely detectable in fat body tissue [68]; however, in other tissues, such as the gut, AQP1 is upregulated. Decreases in AQP in the fat body of *G*. *coquereliana* suggests that AQP may be involved in mechanisms that protect the insects from water loss due to cold temperatures. The data collected in this study differ from those collected in our previous study on cockroaches subjected to 8 h of cold stress and 8 h of recovery time [30]. In this previous research, AQP expression significantly increased in fat body, but it was most likely related to the recovery time of the treatment. Interestingly, the levels of both HSP and AQP in control samples were much higher in fat body than in muscles what may indicate for the important role of fat body tissue in the ability of tropical insects to cope with environmental changes.

Living organisms fit their oxygen-based ATP production to their energy requirements. The main generators of ATP molecules in the cell are mitochondria; thus, their metabolism is particularly interesting during periods of cold stress. Multiple mitochondrial enzymes have been shown to be suppressed during the winter months in both freeze-avoiding *Eurosta scudderiana* and freeze-tolerant *E*. *solidaginis* [69]. Activities of citrate synthase, NAD-isocitrate dehydrogenase and glutamate dedydrogenase were reduced by approximately 50% during the winter months in both of these species [40]. In the silkworm *Bombyx mori*, the activity levels of Krebs cycle enzymes, such as succinate dehydrogenase and malate dehydrogenase, were also significantly lowered [58]. Kukal et al. [70] showed that, in the moth *Gyenaphora groenlandica*, large numbers of mitochondria are reduced following prolonged cold acclimation. In *E*. *solidaginis*, reduced mitochondrial DNA content [42] and decreased cytochrome c oxidase (COX) activity has been observed over the winter months [71].

In our study, we analysed bioenergetics parameters, such as respiratory control ratio (RCR), oxygen consumption in state 3 phosphorylation and state 4 respiration and UCP activity in mitochondria isolated from the muscles and fat body of *G*. *coquereliana*. In both muscle and fat body mitochondrial preparations, we observed decrease in RCR, indicating that there was a lower mitochondrial coupling between state 3 and state 4 respiration (Figs 5 and 6). The drop in RCR in cold-stressed insects was accompanied by a decrease in the rate of phosphorylation in fat body and an increase in state 4 respiration in muscle mitochondria. In addition, we observed a decrease in uncoupling protein activity in muscle mitochondria and an increase in UCP activity in fat body mitochondria. UCPs, proteins that are located in the inner mitochondrial membrane stimulated by free fatty acids (FFA) and inhibited by purine nucleotides (PN) uncouple electron transport through respiratory chain from ATP synthesis, leading to the decrease in the efficiency of ATP synthesis [72].

Uncoupling is the primary mechanism by which the brown adipose tissue of mammals produces heat. Thus, the higher activity of UCP in fat body of insects under cold stress may suggest that UCPs may also play a thermogenic role in insects. This, in turn, suggests important role of fat body tissue in the cold physiology of insects. The possible involvement of UCP4 in heat generation by uncoupled respiration has been previously suggested by Da-Re et al. [48] in the mitochondria of *D*. *melanogaster* larvae.

Colinet [49] showed disruption of ATP synthesis in insect exposed to low temperature and indicated for crucial role of mitochondria in maintenance of energy homeostasis under cold [73]. It could be related to the activation of UCPs, which decrease oxidative phosphorylation yield. In insect mitochondria UCP activity lowered reactive oxygen species (ROS) level which production is enhanced by cold stress [34]. It has been evidenced that in fat body and muscle mitochondria of *G*. *coquereliana* cockroach, activation of UCP decreased level of superoxide anion [47,53], thereby leading to a reduction of its potential damaging effect. Similar, Alves-Bezerra et al. [46] indicate that UCP4 may be involved in an antioxidant mechanisms and protect cells from reactive oxygen species in *Rhodnius prolixus* stressed with cold. Increase of UCP activity may correspond to increased level of HSP 70 which we observed in *G*. *coquereliana*.

Summarizing tropical cockroaches *G*. *coquereliana* experience metabolic changes in fat body and muscle tissue in response to the short (3 h) cold stress, the insect response to cold stress is mainly related to the changes in protein expression as was observed for HSP70, AQP, and activity, as was observed for UCP4, but pattern of these changes differ in both analysed tissues. Moreover, significant changes of bioenergetics parameters were noted in mitochondria isolated from insect exposed to cold indicating regulation of bioenergetics processes according to the insect energy requirements. Taking into account similar function of insect fat body and adipose tissue of mammals, the thermogenic role of fat body in response to cold cannot be excluded. We suspect that UCP4 in fat body might play similar role as thermogenin (UCP1) in brown fat of hibernating animals. However, for better understanding of the possible role of fat body and UCP4 in insect thermoregulation further, detailed studies are required. Our results indicate that aquaporins are engaged in response to cold temperatures not only in insect of subarctic zone, but also in tropical insect. It would be interesting to analyse how the distribution of AQPs between cell membrane and internal membrane systems changes in response to cold and which isoforms play crucial role in protecting tropical cockroaches from cold stress. More studies are needed to follow up on these outstanding questions, especially given that the data of the physiological adaptation of tropical insects to cold temperatures are few.

## Supporting information

S1 Table Raw data. (DOC)

## Abbreviations

AQPs: aquaporins
HSPs: heat shock proteins
UCP4: uncoupling protein 4

## References

[1] Strok NE (2003) Biodiversity In: Encyclopedia of insects. Ed. Resh VH, Cardé RT. Amsterdam, Boston: Academic Press pp. 85–91.

[2] Bale JS (1996) Insect cold hardiness: A matter of life and death. Eur J Entomol 93: 369–382.

[3] Clark MS, Worland MR (2008) How insects survive the cold: molecular mechanisms-a review. J Comp Physiol B 178: 917–933. 10.1007/s00360-008-0286-4 18584182

[4] Denlinger DL, Lee RE (2010) Low temperature biology in insects: Cam Univ Press.

[5] Lee RE, Denlinger DL (1991) Insects at low temperature. New York: Chapman and Hall x, 513 p. p.

[6] Doucet D, Walker VK, Qin W (2009) The bugs that came in from the cold: molecular adaptations to low temperatures in insects. Cell Mol Life Sci 66: 1404–1418. 10.1007/s00018-009-8320-6 19129970 PMC11131528

[7] Storey KB, Storey JM (2013) Molecular biology of freezing tolerance. Compr Physiol 3: 1283–1308. 10.1002/cphy.c130007 23897687

[8] Joanisse DR, Storey KB (1995) Temperature acclimation and seasonal responses by enzymes in cold-hardy gall insects. Arch Insect Biochem Physiol 28: 339–349.

[9] Teets NM, Peyton JT, Ragland GJ, Colinet H, Renault D, Hahn DA, et al (2012) Combined transcriptomic and metabolomic approach uncovers molecular mechanisms of cold tolerance in a temperate flesh fly. Physiol Genomics 44: 764–777. 10.1152/physiolgenomics.00042.2012 22735925

[10] Khani A, Moharramipour S (2010) Cold hardiness and supercooling capacity in the overwintering larvae of the codling moth, *Cydia pomonella*. J Insect Sci 10: 83 10.1673/031.010.8301 20673068 PMC3383407

[11] Rozsypal J, Kostal V, Zahradnickova H, Simek P (2013) Overwintering strategy and mechanisms of cold tolerance in the codling moth (*Cydia pomonella*). PLoS One 8: e61745 10.1371/journal.pone.0061745 23613923 PMC3629207

[12] Walters KR, Pan QF, Serianni AS, Duman JG (2009) Cryoprotectant biosynthesis and the selective accumulation of threitol in the freeze-tolerant Alaskan beetle, *Upis ceramboides*. J Biol Chem 284: 16822–16831. 10.1074/jbc.M109.013870 19403530 PMC2719318

[13] Wharton DA, Pow B, Kristensen M, Ramlov H, Marshall CJ (2009) Ice-active proteins and cryoprotectants from the New Zealand alpine cockroach, *Celatoblatta quinquemaculata*. J Insect Physiol 55: 27–31. 10.1016/j.jinsphys.2008.09.007 18955061

[14] Campbell EM, Ball A, Hoppler S, Bowman AS (2008) Invertebrate aquaporins: a review. J Comp Physiol B 178: 935–955. 10.1007/s00360-008-0288-2 18594835

[15] Philip BN, Yi SX, Elnitsky MA, Lee RE Jr. (2008) Aquaporins play a role in desiccation and freeze tolerance in larvae of the goldenrod gall fly, *Eurosta solidaginis*. J Exp Biol 211: 1114–1119. 10.1242/jeb.016758 18344486

[16] Tomkowiak E, Pienkowska J (2009) The current knowledge of invertebrate aquaporin water channels with particular emphasis on insect AQPs. Post Biol Kom 39: 203–216.

[17] Yi W, Zhang Y, Tian Y, Guo J, Li Y, Guo A (2013) A subset of cholinergic mushroom body neurons requires G_0_ signaling to regulate sleep in *Drosophila*. Sleep 36: 1809–1821. 10.5665/sleep.3206 24293755 PMC3825430

[18] Duman JG (2001) Antifreeze and ice nucleator proteins in terrestrial arthropods. Annu Rev Physiol 63: 327–357. 10.1146/annurev.physiol.63.1.327 11181959

[19] Levis NA, Yi SX, Lee RE Jr. (2012) Mild desiccation rapidly increases freeze tolerance of the goldenrod gall fly, *Eurosta solidaginis*: evidence for drought-induced rapid cold-hardening. J Exp Biol 215: 3768–3773. 10.1242/jeb.076885 22899523

[20] Zachariassen KE, Kristiansen E (2000) Ice nucleation and antinucleation in nature. Cryobiology 41: 257–279. 10.1006/cryo.2000.2289 11222024

[21] Davies PL, Baardsnes J, Kuiper MJ, Walker VK (2002) Structure and function of antifreeze proteins. Philos Trans R Soc Lond B Biol Sci 357: 927–935. 10.1098/rstb.2002.1081 12171656 PMC1692999

[22] Duman JG, Bennett V, Sformo T, Hochstrasser R, Barnes BM (2004) Antifreeze proteins in Alaskan insects and spiders. J Insect Physiol 50: 259–266. 10.1016/j.jinsphys.2003.12.003 15081818

[23] Qin W, Doucet D, Tyshenko MG, Walker VK (2007) Transcription of antifreeze protein genes in *Choristoneura fumiferana*. Insect Mol Biol 16: 423–434. 10.1111/j.1365-2583.2007.00743.x 17651234

[24] Benoit JB, Lopez-Martinez G, Teets NM, Phillips SA, Denlinger DL (2009) Responses of the bed bug, *Cimex lectularius*, to temperature extremes and dehydration: levels of tolerance, rapid cold hardening and expression of heat shock proteins. Med Vet Entomol 23: 418–425. 10.1111/j.1365-2915.2009.00832.x 19941608

[25] Hayward SA, Rinehart JP, Denlinger DL (2004) Desiccation and rehydration elicit distinct heat shock protein transcript responses in flesh fly pupae. J Exp Biol 207: 963–971. 14766955 10.1242/jeb.00842

[26] Lyytinen A, Mappes J, Lindstrom L (2012) Variation in Hsp70 levels after cold shock: signs of evolutionary responses to thermal selection among *Leptinotarsa decemlineata* populations. PLoS One 7: e31446 10.1371/journal.pone.0031446 22319631 PMC3271087

[27] Hanson RW, Reshef L (1997) Regulation of phosphoenolpyruvate carboxykinase (GTP) gene expression. Annu Rev Biochem 66: 581–611. 10.1146/annurev.biochem.66.1.581 9242918

[28] Somme L (1999) The physiology of cold hardiness in terrestrial arthropods. Eur J Entomol 96: 1–10.

[29] Zhou XR, Li YY, Li N, Pang BP (2015) Relationship between supercooling capability and cryoprotectant content in eggs of *Pararcyptera microptera* meridionalis (Orthoptera: Acrypteridae). Cryo Letters 36: 270–277. 26576002

[30] Chowański S, Lubawy J, Spochacz M, Paluch E, Smykalla G, Rosiński G, et al (2015) Cold induced changes in lipid, protein and carbohydrate levels in the tropical insect *Gromphadorhina coquereliana*. Comp Biochem Physiol A Mol Integr Physiol 183: 57–63. 10.1016/j.cbpa.2015.01.007 25624163

[31] Izumi Y, Sonoda S, Yoshida H, Danks HV, Tsumuki H (2006) Role of membrane transport of water and glycerol in the freeze tolerance of the rice stem borer, Chilo suppressalis Walker (Lepidoptera: Pyralidae). J Insect Physiol 52: 215–220. 10.1016/j.jinsphys.2005.11.001 16359699

[32] Zelenina M, Tritto S, Bondar AA, Zelenin S, Aperia A (2004) Copper inhibits the water and glycerol permeability of aquaporin-3. J Biol Chem 279: 51939–51943. 10.1074/jbc.M407645200 15456785

[33] Clark MS, Thorne MA, Purac J, Burns G, Hillyard G, Popovic ZD, et al (2009) Surviving the cold: molecular analyses of insect cryoprotective dehydration in the Arctic springtail *Megaphorura arctica* (Tullberg). BMC Genomics 10: 328 10.1186/1471-2164-10-328 19622137 PMC2726227

[34] Lalouette L, Williams CM, Hervant F, Sinclair BJ, Renault D (2011) Metabolic rate and oxidative stress in insects exposed to low temperature thermal fluctuations. Comp Biochem Physiol A Mol Integr Physiol 158: 229–234. 10.1016/j.cbpa.2010.11.007 21074633

[35] Thorne MAS, Worland MR, Feret R, Deery MJ, Lilley KS, Clark MS (2011) Proteomics of cryoprotective dehydration in *Megaphorura arctica* Tullberg 1876 (Onychiuridae: Collembola). Insect Mol Biol 20: 303–310. 10.1111/j.1365-2583.2010.01062.x 21199019

[36] Kayukawa T, Ishikawa Y (2009) Chaperonin contributes to cold hardiness of the onion maggot *Delia antiqua* through repression of depolymerization of actin at low temperatures. PLoS One 4: e8277 10.1371/journal.pone.0008277 20011606 PMC2788269

[37] Zhang GJ, Storey JM, Storey KB (2011) Chaperone proteins and winter survival by a freeze tolerant insect. J Insect Physiol 57: 1115–1122. 10.1016/j.jinsphys.2011.02.016 21382374

[38] Urbański A, Czarniewska E, Baraniak E, Rosiński G (2014) Developmental changes in cellular and humoral responses of the burying beetle *Nicrophorus vespilloides* (Coleoptera, Silphidae). J Insect Physiol 60: 98–103. 10.1016/j.jinsphys.2013.11.009 24295868

[39] Urbański A, Czarniewska E, Baraniak E, Rosiński G (2016) Impact of cold on the immune system of burying beetle, *Nicrophorus vespilloides* (Coleoptera: Silphidae). Insect Sci. 10.1111/1744-7917.12321 26799536

[40] Joanisse DR, Storey KB (1994) Mitochondrial enzymes during overwintering in 2 species of cold-hardy gall insects. Insect Biochem Mol Biol 24: 145–150.

[41] Joanisse DR, Storey KB (1996) Fatty acid content and enzymes of fatty acid metabolism in overwintering cold-hardy gall insects. Physiol Zool 69: 1079–1095.

[42] Levin DB, Danks HV, Barber SA (2003) Variations in mitochondrial DNA and gene transcription in freezing-tolerant larvae of *Eurosta solidaginis* (Diptera: Tephritidae) and *Gynaephora groenlandica* (Lepidoptera: Lymantriidae). Insect Mol Biol 12: 281–289. 12752662 10.1046/j.1365-2583.2003.00413.x

[43] Vences M, Wollenberg KC, Vieites DR, Lees DC (2009) Madagascar as a model region of species diversification. Trends Ecol Evol 24: 456–465. 10.1016/j.tree.2009.03.011 19500874

[44] WheaterSpark (2015) Historical Weather For 2014 in Antanaagrave;naraigrave;vo, Madagascar—WeatherSpark; https://weatherspark.com/history/29102/2014/Antananarivo-Antananarivo-Madagascar. pp. WheaterSpark: beautiful weather graphs and maps making in-depth weather information easily accessible.

[45] Airaksinen S, Jokilehto T, Rabergh CM, Nikinmaa M (2003) Heat- and cold-inducible regulation of HSP70 expression in zebrafish ZF4 cells. Comp Biochem Physiol B Biochem Mol Biol 136: 275–282. 14529753 10.1016/s1096-4959(03)00205-7

[46] Alves-Bezerra M, Cosentino-Gomes D, Vieira LP, Rocco-Machado N, Gondim KC, Meyer-Fernandes JR (2014) Identification of uncoupling protein 4 from the blood-sucking insect *Rhodnius prolixus* and its possible role on protection against oxidative stress. Insect Biochem Mol Biol 50: 24–33. 10.1016/j.ibmb.2014.03.011 24746771

[47] Slocinska M, Rosinski G, Jarmuszkiewicz W (2016) Activation of mitochondrial uncoupling protein 4 and ATP-sensitive potassium channel cumulatively decreases superoxide production in insect mitochondria. Protein Pept Lett 23: 63–68. 26548865 10.2174/0929866523666151106121943

[48] Da-Re C, De Pitta C, Zordan MA, Teza G, Nestola F, Zeviani M, et al (2014) UCP4C mediates uncoupled respiration in larvae of *Drosophila melanogaster*. EMBO Rep 15: 586–591. 10.1002/embr.201337972 24639557 PMC4210097

[49] Colinet H (2011) Disruption of ATP homeostasis during chronic cold stress and recovery in the chill susceptible beetle (*Alphitobius diaperinus*). Comp Biochem Physiol A Mol Integr Physiol 160: 63–67. 10.1016/j.cbpa.2011.05.003 21596153

[50] Garcia-Garcia E, Garcia-Garcia PL, Rosales C (2009) An fMLP receptor is involved in activation of phagocytosis by hemocytes from specific insect species. Dev Comp Immunol 33: 728–739. 10.1016/j.dci.2008.12.006 19166874

[51] Dubois M, Gilles KA, Hamilton JK, Rebers PA, Smith F (1956) Colorimetric method for determination of sugars and related substances. Anal Chem 28: 350–356.

[52] Folch J, Lees M, Stanley GHS (1957) A simple method for the isolation and purification of total lipides from animal tissues. J Biol Chem 226: 497–509. 13428781

[53] Słocińska M, Antos-Krzemińska N, Rosiński G, Jarmuszkiewicz W (2011) Identification and characterization of uncoupling protein 4 in fat body and muscle mitochondria from the cockroach *Gromphadorhina cocquereliana*. J Bioenerg Biomembr 43: 717–727. 10.1007/s10863-011-9385-0 21997226

[54] Słocińska M, Antos-Krzeminska N, Gołębiowski M, Kuczer M, Stępnowski P, Rosiński G, et al (2013) UCP4 expression changes in larval and pupal fat bodies of the beetle *Zophobas atratus* under adipokinetic hormone treatment. Comp Biochem Physiol A Mol Integr Physiol 166: 52–59. 10.1016/j.cbpa.2013.05.009 23688504

[55] Sujak P (1984) Oxidative metabolism in the fat body during the life cycle of insects. Poznań: Adam Mickiewicz University Press.

[56] Overgaard J, Malmendal A, Sorensen JG, Bundy JG, Loeschcke V, Nielsen NC, et al (2007) Metabolomic profiling of rapid cold hardening and cold shock in *Drosophila melanogaster*. J Insect Physiol 53: 1218–1232. 10.1016/j.jinsphys.2007.06.012 17662301

[57] Tamang AM, Kalra B, Parkash R (2017) Cold and desiccation stress induced changes in the accumulation and utilization of proline and trehalose in seasonal populations of *Drosophila immigrans*. Comp Biochem Physiol A Mol Integr Physiol 203: 304–313. 10.1016/j.cbpa.2016.10.011 27793614

[58] Singh A, Jaiswal SK, Sharma B (2013) Low temperature induced stress and biomolecular imbalances in insects with special reference to silkworms. J Biochem Res 1: 26–35.

[59] Michaud MR (2007) Molecular physiology of insect low temperature stress responses. Ohio, USA: The Ohio State University.

[60] Feder ME, Hofmann GE (1999) Heat-shock proteins, molecular chaperones, and the stress response: evolutionary and ecological physiology. Annu Rev Physiol 61: 243–282. 10.1146/annurev.physiol.61.1.243 10099689

[61] Rinehart JP, Denlinger DL (2000) Heat-shock protein 90 is down-regulated during pupal diapause in the flesh fly, *Sarcophaga crassipalpis*, but remains responsive to thermal stress. Insect Mol Biol 9: 641–645. 11122473 10.1046/j.1365-2583.2000.00230.x

[62] Yocum GD, Joplin KH, Denlinger DL (1998) Upregulation of a 23 kDa small heat shock protein transcript during pupal diapause in the flesh fly, *Sarcophaga*, *crassipalpis*. Insect Biochem Mol Biol 28: 677–682. 9755478 10.1016/s0965-1748(98)00046-0

[63] Colinet H, Lee SF, Hoffmann A (2010) Temporal expression of heat shock genes during cold stress and recovery from chill coma in adult *Drosophila melanogaster*. FEBS J 277: 174–185. 10.1111/j.1742-4658.2009.07470.x 19968716

[64] Yocum GD (2001) Differential expression of two HSP70 transcripts in response to cold shock, thermoperiod, and adult diapause in the Colorado potato beetle. J Insect Physiol 47: 1139–1145. 12770192 10.1016/s0022-1910(01)00095-6

[65] Foster NL, Lukowiak K, Henry TB (2015) Time-related expression profiles for heat shock protein gene transcripts (HSP40, HSP70) in the central nervous system of *Lymnaea stagnalis* exposed to thermal stress. Commun Integr Biol 8: e1040954 10.1080/19420889.2015.1040954 26478775 PMC4594255

[66] Cohen E (2012) Roles of aquaporins in osmoregulation, desiccation and cold hardiness in insects. Entomology, Ornithology & Herpetology S1:001: 1–17.

[67] Yi SX, Benoit JB, Elnitsky MA, Kaufmann N, Brodsky JL, Zeidel ML, et al (2011) Function and immuno-localization of aquaporins in the Antarctic midge *Belgica antarctica*. J Insect Physiol 57: 1096–1105. 10.1016/j.jinsphys.2011.02.006 21315725 PMC8875278

[68] Philip BN, Kiss AJ, Lee RE Jr. (2011) The protective role of aquaporins in the freeze-tolerant insect *Eurosta solidaginis*: functional characterization and tissue abundance of EsAQP1. J Exp Biol 214: 848–857. 10.1242/jeb.051276 21307072

[69] Storey JM, Storey KB (1985) Freezing and cellular metabolism in the gall fly larva, *Eurosta solidaginis*. J Comp Physiol B 155: 333–337.

[70] Kukal O, Duman JG, Serianni AS (1989) Cold-induced mitochondrial degradation and cryoprotectant synthesis in freeze-tolerant arctic caterpillars. J Comp Physiol B 158: 661–671. 2715455 10.1007/BF00693004

[71] McMullen DC, Storey KB (2008) Suppression of Na^+^K^+^-ATPase activity by reversible phosphorylation over the winter in a freeze-tolerant insect. J Insect Physiol 54: 1023–1027. 10.1016/j.jinsphys.2008.04.001 18501921

[72] Sluse FE, Jarmuszkiewicz W, Navet R, Douette P, Mathy G, Sluse-Goffart CM (2006) Mitochondrial UCPs: new insights into regulation and impact. Biochim Biophys Acta 1757: 480–485. 10.1016/j.bbabio.2006.02.004 16597432

[73] Colinet H, Renault D, Roussel D (2016) Cold acclimation allows *Drosophila* flies to maintain mitochondrial functioning under cold stress. Insect Biochem Mol Biol 80: 52–60. 10.1016/j.ibmb.2016.11.007 27903433

