# Supplementary material for: The physiological role of fat body and muscle tissues in response to cold stress in the tropical cockroach Gromphadorhina coquereliana
Source: PLoS One. 2017 Mar 2;12(3):e0173100. doi: 10.1371/journal.pone.0173100 (PMC5333868; doi:10.1371/journal.pone.0173100)
Supplement: S1 Table — (DOC) [file pone.0173100.s001.doc]

**Table 1. Level of HSP70 and AQPs in fat body and leg muscles of control (2 °C) and tested (4 °C; 3 h) cockroaches.**

|  | **HSP70 level in fat body [a.u.]** | | **HSP70 level in muscles [a.u.]** | | **AQPs level in fat body [a.u.]** | | **AQPs level in muscles [a.u.]** | |
| --- | --- | --- | --- | --- | --- | --- | --- | --- |
|  | **control** | **cold** | **control** | **cold** | **control** | **cold** | **control** | **cold** |
|  | 332,3 | 674,8 | 200,6 | 310,8 | 194,1 | 118,2 | 115,4 | 49,8 |
|  | 379,4 | 820,1 | 233,7 | 326,5 | 205,4 | 120,3 | 116,1 | 89,6 |
|  | 423,3 | 834,5 | 246,2 | 330,1 | 211,3 | 122,7 | 135,5 | 106,1 |
|  | 464,5 | 932,6 | 283,7 | 333,7 | 222,1 | 128,1 | 147,5 | 108,2 |
|  |  |  |  |  |  | 131,5 |  | 115,2 |
| ***n*** | 4 | 4 | 4 | 4 | 4 | 5 | 4 | 5 |
| **mean** | 399,9 | 815,5 | 241,1 | 325,3 | 208,2 | 124,2 | 128,6 | 93,78 |
| **SD** | 56,89 | 106,33 | 34,33 | 10,09 | 11,68 | 5,52 | 15,66 | 26,32 |
| **SEM** | 28,45 | 53,14 | 17,17 | 5,04 | 5,84 | 2,47 | 7,83 | 11,77 |
| ***t*** | 6,8950 | | 4,7080 | | 14,3800 | | 2,3210 | |
| ***p*** | 0,0005 | | 0,0033 | | < 0,0001 | | 0,0533 | |

**Table 2. Content of glycogen, total lipids and soluble proteins in fat body and leg muscles of control (2 °C) and tested (4 °C; 3 h) cockroaches.**

|  | **glycogen content in fat body [μg/mg of dry tissue]** | | **glycogen in muscles [μg/mg of dry tissue]** | | **total lipids in fat body [μg/mg of dry tissue]** | | **total lipids in muscles [μg/mg of dry tissue]** | | **soluble proteins in fat body [μg/mg of dry tissue]** | | **soluble proteins in muscles [μg/mg of dry tissue]** | |
| --- | --- | --- | --- | --- | --- | --- | --- | --- | --- | --- | --- | --- |
|  | **control** | **cold** | **control** | **cold** | **control** | **cold** | **control** | **cold** | **control** | **cold** | **control** | **cold** |
|  | 1,5 | 1,1 | 10,9 | 18,0 | 149,2 | 282,6 | 30,6 | 40,7 | 15,9 | 14,4 | 89,6 | 224,5 |
|  | 2,2 | 2,2 | 17,3 | 18,4 | 180,3 | 305,1 | 37,3 | 43,6 | 20,3 | 21,4 | 125,0 | 230,9 |
|  | 3,7 | 3,6 | 18,3 | 18,9 | 264,2 | 362,5 | 45,9 | 49,2 | 28,7 | 21,8 | 139,3 | 243,1 |
|  | 6,1 | 3,8 | 18,5 | 20,4 | 277,6 | 397,3 | 56,2 | 52,9 | 30,3 | 22,0 | 221,5 | 255,0 |
|  | 8,1 | 4,3 | 23,2 | 22,1 | 396,1 | 492,5 | 56,5 | 53,8 | 31,2 | 22,3 | 224,3 | 255,5 |
|  | 8,1 | 5,2 | 24,5 | 23,6 | 401,4 | 516,4 | 59,5 | 57,0 | 31,8 | 23,7 | 229,0 | 262,4 |
|  | 12,2 | 8,4 | 26,8 | 23,8 | 429,5 | 529,2 | 61,9 | 57,1 | 36,8 | 25,9 | 249,0 | 266,5 |
|  | 17,5 | 10,3 | 27,8 | 25,1 | 478,6 | 585,4 | 65,6 | 65,2 | 36,9 | 31,2 | 261,6 | 267,6 |
|  | 17,6 | 12,4 | 29,3 | 28,9 | 505,5 | 590,0 | 66,7 | 73,7 | 52,5 | 31,9 | 276,2 | 273,7 |
|  | 19,6 | 13,8 | 37,9 | 30,0 | 542,0 | 611,6 | 67,4 | 76,2 | 62,6 | 39,4 | 312,4 | 292,8 |
|  | 22,9 | 14,1 | 44,9 | 30,2 | 557,4 | 617,4 | 83,5 | 77,8 | 67,1 | 47,3 | 312,8 | 303,3 |
|  |  | 14,6 | 46,2 |  | 574,7 | 674,5 | 90,0 | 82,5 | 71,0 | 60,7 | 324,0 | 330,1 |
|  |  | 15,6 |  |  | 578,5 |  |  | 93,4 |  |  | 327,0 |  |
|  |  |  |  |  |  |  |  |  |  |  | 357,7 |  |
| ***n*** | 11 | 13 | 12 | 11 | 13 | 12 | 12 | 13 | 12 | 12 | 14 | 12 |
| **mean** | 10,9 | 8,4 | 27,1 | 23,6 | 410,4 | 497,0 | 60,1 | 63,3 | 40,4 | 30,2 | 246,4 | 267,1 |
| **SD** | 7,51 | 5,28 | 11,02 | 4,56 | 149,60 | 130,70 | 17,04 | 16,17 | 18,37 | 13,14 | 81,81 | 30,13 |
| **SEM** | 2,27 | 1,46 | 3,18 | 1,38 | 41,50 | 37,72 | 4,92 | 4,49 | 5,30 | 3,79 | 21,86 | 8,70 |
| ***t*** | 0,9349 | | 0,9924 | | 1,5360 | | 0,4854 | | 1,5750 | | 0,8290 | |
| ***p*** | 0,3600 | | 0,3323 | | 0,1381 | | 0,6320 | | 0,1295 | | 0,4153 | |

**Table 3. Mitochondrial respiratory activity of mitochondria isolated from fat body and leg muscles of control (2 °C) and tested (4 °C; 3 h) cockroaches.**

|  | **state 4 respiration in fat body [nmoles O_2_/min/g of protein]** | | **state 4 respiration in muscles [nmoles O_2_/min/g of protein]** | | **state 3 respiration in fat body [nmoles O_2_/min/g of protein]** | | **state 3 respiration in muscles [nmoles O_2_/min/g of protein]** | | **RCR in fat body** | | **RCR in muscles** | |
| --- | --- | --- | --- | --- | --- | --- | --- | --- | --- | --- | --- | --- |
|  | **control** | **cold** | **control** | **cold** | **control** | **cold** | **control** | **cold** | **control** | **cold** | **control** | **cold** |
|  | 27,4 | 31,5 | 13,2 | 25,5 | 87,6 | 63,6 | 52,9 | 49,4 | 2,3 | 1,9 | 2,7 | 1,7 |
|  | 27,3 | 31,4 | 14,1 | 25,5 | 96,0 | 64,0 | 58,6 | 52,0 | 2,3 | 1,9 | 2,8 | 1,8 |
|  | 27,3 | 40,0 | 17,4 | 26,8 | 104,0 | 69,1 | 69,8 | 59,5 | 2,8 | 2,1 | 3,5 | 1,9 |
|  | 37,0 | 41,5 | 22,0 | 30,4 | 106,8 | 77,0 | 76,8 | 62,3 | 3,3 | 2,5 | 4,0 | 2,1 |
|  | 44,0 | 41,5 | 26,9 | 40,1 | 110,4 | 77,3 | 77,0 | 82,3 | 3,8 | 2,6 | 4,0 | 2,3 |
|  |  |  | 27,9 | 45,5 | 122,0 | 82,9 | 104,7 | 82,4 |  |  | 4,1 | 2,4 |
|  |  |  | 38,4 | 59,0 |  |  | 110,4 | 106,9 |  |  | 4,1 | 2,7 |
|  |  |  |  |  |  |  | 110,4 | 201,0 |  |  | 5,0 | 3,4 |
| *n* | 5 | 5 | 7 | 7 | 6 | 6 | 8 | 8 | 5 | 5 | 8 | 8 |
| mean | 32,6 | 37,2 | 22,9 | 36,1 | 104,5 | 72,3 | 82,6 | 87,0 | 2,9 | 2,2 | 3,8 | 2,3 |
| SD | 7,60 | 5,24 | 8,97 | 12,74 | 11,87 | 7,94 | 23,06 | 49,91 | 0,63 | 0,33 | 0,76 | 0,57 |
| SEM | 3,40 | 2,34 | 3,39 | 4,82 | 4,84 | 3,24 | 8,15 | 17,64 | 0,28 | 0,14 | 0,26 | 0,20 |
| *t* | 1,1010 | | 2,2460 | | 5,5150 | | 0,2288 | | 2,1930 | | 4,4150 | |
| *p* | 0,3030 | | 0,0443 | | 0,0003 | | 0,8224 | | 0,0596 | | 0,0006 | |

Table 4. Activity of UCP protein in mitochondria of fat body and leg muscles of control (2 °C) and tested (4 °C; 3 h) cockroaches.

|  | **UCP activity in fat body [%]** | | | | **UCP activity in muscles [%]** | | | |
| --- | --- | --- | --- | --- | --- | --- | --- | --- |
|  | **in presence of PA** | | **in presence of GTP** | | **in presence of PA** | | **in presence of GTP** | |
|  | **control** | **cold** | **control** | **cold** | **control** | **cold** | **control** | **cold** |
|  | 63 | 80 | 14 | 20 | 120 | 48 | 15 | 20 |
|  | 63 | 85 | 20 | 24 | 133 | 48 | 20 | 24 |
|  | 72 | 86 | 20 | 24 | 133 | 62 | 22 | 25 |
|  | 80 | 90 | 25 | 30 | 150 | 70 | 25 | 30 |
| ***n*** | 4 | 4 | 4 | 4 | 4 | 4 | 4 | 4 |
| **mean** | 69,5 | 85,25 | 19,75 | 24,5 | 134 | 57 | 20,5 | 24,75 |
| **SD** | 8,19 | 4,11 | 4,50 | 4,12 | 12,30 | 10,89 | 4,20 | 4,11 |
| **SEM** | 4,09 | 2,06 | 2,25 | 2,06 | 6,15 | 5,45 | 2,10 | 2,06 |
| ***t*** | 3,4390 | | 1,5570 | | 9,3720 | | 1,4450 | |
| ***p*** | 0,0138 | | 0,1706 | | < 0,0001 | | 0,1985 | |
